# Solid base catalysed 5-HMF oxidation to 2,5-FDCA over Au/hydrotalcites: fact or fiction?†
†Electronic supplementary information (ESI) available: Catalyst synthesis, additional characterisation and reaction conditions. See DOI: 10.1039/c5sc00854a


**DOI:** 10.1039/c5sc00854a

**Published:** 2015-06-08

**Authors:** Leandro Ardemani, Giannantonio Cibin, Andrew J. Dent, Mark A. Isaacs, Georgios Kyriakou, Adam F. Lee, Christopher M. A. Parlett, Stephen A. Parry, Karen Wilson

**Affiliations:** a European Bioenergy Research Institute , Aston University , Birmingham B4 7ET , UK . Email: a.f.lee@aston.ac.uk; b Diamond Light Source , Harwell Science and Innovation Campus , Didcot OX11 0DE , UK; c Department of Chemistry , University of Hull , Hull , HU6 7RX , UK

## Abstract

Nanoparticulate gold has emerged as a promising catalyst for diverse mild and efficient selective aerobic oxidations. However, the mechanism of such atom-economical transformations, and synergy with functional supports, remains poorly understood. Alkali-free Mg–Al hydrotalcites are excellent solid base catalysts for the aerobic oxidation of 5-hydroxymethylfurfural (HMF) to 2,5-furan dicarboxylic acid (FDCA), but only in concert with high concentrations of metallic gold nanoparticles. In the absence of soluble base, competitive adsorption between strongly-bound HMF and reactively-formed oxidation intermediates site-blocks gold. Aqueous NaOH dramatically promotes solution phase HMF activation, liberating free gold sites able to activate the alcohol function within the metastable 5-hydroxymethyl-2-furancarboxylic acid (HMFCA) reactive intermediate. Synergistic effects between moderate strength base sites within alkali-free hydrotalcites and high gold surface concentrations can afford highly selective and entirely heterogeneous catalysts for aqueous phase aldehyde and alcohol cascade oxidations pertinent to biomass transformation.

## Abstract 2

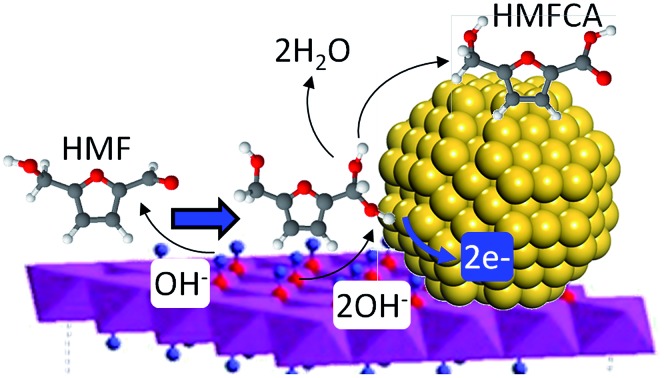
Synergistic effects between alkali-free hydrotalcites and gold nanoparticles afford efficient heterogeneous catalysts for the cascade oxidation of 5-HMF to 2,5-FDCA.

## 

Biomass offers the most readily implemented, and low cost, sustainable ‘drop-in’ alternative to existing fossil fuel-derived transportation fuels,^1^ and the only non-petroleum route to organic molecules essential to the manufacture of bulk, fine and speciality chemicals and polymers^2^ required to meet future societal demands.^3^,^4^ The development of biorefineries offering the co-production of fuels, chemicals and energy,^5^ analogous to current petroleum refineries which deliver high volume/low value (fuels and commodity chemicals) and low volume/high value (fine/speciality chemicals) products, will underpin commercial exploitation of the biomass feedstocks. In this respect, the US DoE has identified 12 platform chemicals obtainable through sugars *via* the (bio)chemical transformation of lignocellulosic biomass. 2,5-Furan dicarboxylic acid (FDCA) is one of the key near market platform chemicals with an estimated value of $50.5 billion, and is viewed as a potential replacement for terephthalic acid in various polyesters and an intermediate to other polymers, fine chemicals, pharmaceuticals and agrochemicals.^6^–^10^ Heterogeneous catalysis and process engineering are pivotal to realizing the potential of lignocellulosic biomass for the production of such renewable chemicals^11^–^16^ underpinned by the rational design of multifunctional tailored catalysts able to affect cascade and telescopic reactions at mild temperatures in the aqueous phase.

A promising route to FDCA is *via* the aerobic selective oxidation of 5-hydroxymethylfurfural (HMF), which in turn may be derived from cellulose through isomerisation/dehydration of hexoses.^17^–^20^ The stepwise heterogeneously catalysed transformation of HMF to FDCA using molecular oxygen is highly desirable,^8^ obviating the need for stoichiometric oxidants such as KMnO_4_ or homogeneous metal halides (Co/Mn/Br)^21^ which necessitate harsh (70 bar) and corrosive conditions and poor atom efficiencies due to significant waste generation during product purification. In the past five years, gold nanoparticles supported on reducible metal oxides (Au/TiO_2_,^6^ Au/CeO_2_,^7^ Au–Cu/TiO_2_ (ref. 22) and Au/Ce_1–*x*_Bi_*x*_O_2–*δ*_ (ref. 23)) have been reported to catalyse the aerobic oxidation of HMF to FDCA, but require the addition of 1–20 equiv. soluble base to accelerate alkoxide formation and C–H activation in the catalytic cycle. Indeed, a requirement for base addition has emerged as a general phenomenon in gold catalysed alcohol oxidations,^24^,^25^ and processing the attendant aqueous waste stream resulting from subsequent acidification and purification of the reaction mixture^8^ presents a serious barrier to commercialization.^26^ A recent report suggests that bimetallic AuPd nanoparticles supported on carbon nanotubes may obviate the requirement for basicity, although reaction is slow and proceeds under high pO_2_.^27^ Solid base supports may circumvent the need for liquid base addition,^28^–^30^ with Au/Mg–Al hydrotalcites (HT) reported as effective for FDCA production from HMF under an atmospheric oxygen pressure,^31^ or using a biphasic solvent system over AuPd/HT albeit it in the presence of additional Na_2_CO_3_.^32^ However, despite the topical nature of Au catalysis, the role of basic supports in aqueous phase oxidations,^33^ and potential contribution of homogeneous base on observed performance,^24^,^34^,^35^ remains hotly debated.^25^,^33^,^36^–^39^ This in part reflects the synthetic methodologies often employed to synthesise inorganic basic supports such as hydrotalcites, notably precipitation with Na_2_CO_3_/NaOH,^31^ which result in contamination by soluble alkali residues.^40^–^42^ This uncertainty in turn hampers elucidation of the role played by basic supports within catalytic oxidation cycles.

Herein we show that the selective aerobic oxidation of HMF to FDCA over gold nanoparticles on an alkali-free hydrotalcite carrier exhibits unusual and unexpected sensitivity to the surface concentration of metallic gold. Specifically, kinetic and *operando* spectroscopic studies reveal that low concentrations of surface gold require liquid base in order to overcome rate-limiting competitive adsorption and effect oxidation of reaction intermediates. In contrast, high gold concentrations can achieve high yields of FDCA over a solid base alone. This interplay between reactants and intermediates at different reaction centres within bifunctional catalysts has profound implications for heterogeneously catalysed cascades.

To perform a comprehensive study, a series of Au/HT catalyst materials were prepared using an alkali-free co-precipitation of the Mg–Al hydrotalcite support,^43^ and subsequent deposition-precipitation of HAuCl_4_. In order to optimise the catalyst synthesis, thermal evolution of the gold precursor was investigated by *in situ* Au L_III_ X-ray absorption near edge spectroscopy (XANES) (Fig. 1). XANES spectra of the as-prepared materials are consistent with the presence of a Au(iii) salt, possibly Au(NH_*x*_)_*y*_(OH)_*z*_^*n*–^ which may form during deposition-precipitation with ammonia or urea,^44^,^45^ and ∼10% of Au(OH)_3_. Heating to 65 °C under flowing air initiated precursor decomposition and the concomitant appearance of Au_2_O_3_, which remained stable to ∼110 °C before decomposing to metallic Au. Complete decomposition of both the Au(NH_*x*_)_*y*_(OH)_*z*_^*n*–^ precursor and Au_2_O_3_ to metallic gold required calcination >170 °C. The corresponding Extended X-ray Absorption Fine Structure (EXAFS) for a 2 wt% Au/HT calcined at 200 °C yielded a nearest neighbour Au–Au coordination of 10.6 (Fig. S1 and Table S1†), consistent with 4.2 nm diameter metal nanoparticles,^46^,^47^ in agreement with HRTEM, XRD and XPS (Fig. S2–S4†).

**Fig. 1:**
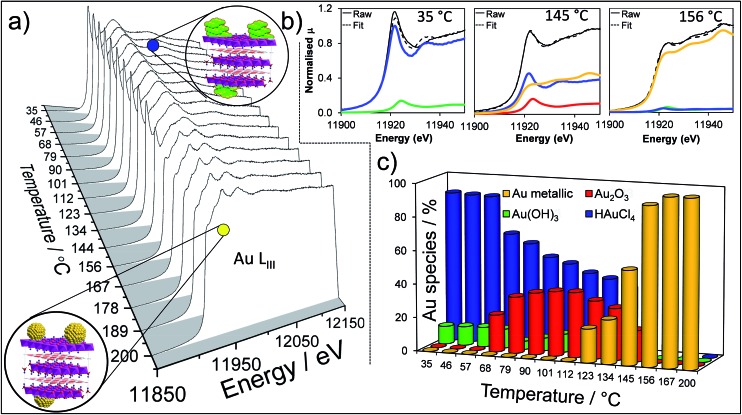
*In situ* Au L_III_ XANES during thermal processing of the HAuCl_4_/Mg–Al HT precursor: (a) thermal evolution of normalised XANES spectra; (b) representative least squares fitted XANES spectra to reference gold species; (c) quantitative thermal evolution of fitted Au species.

Atmospheric pressure HMF aerobic oxidation over the resultant 200 °C calcined 2 wt% Au/HT *in the absence of additional NaOH* proceeded efficiently (conversions >80%, Fig. 2), but was only selective to 5-hydroxymethyl-2-furancarboxylic acid (HMFCA) resulting from oxidation of the carbonyl function.† Further oxidation to 5-formyl-2-furan carboxylic acid (FFCA) and the desired FDCA product was slow, contrary to an earlier report using a comparable 2 wt% Au catalyst prepared on an *alkali-precipitated* Mg–Al HT, wherein near quantitative yields of FDCA were reported under identical reaction conditions.^31^ This discrepancy highlights an important role for soluble base in activating R–OH functions over Au, arising either from alkali contaminants leached from Na_2_CO_3_/NaOH precipitated hydrotalcites, or partially soluble brucite co-existing with the hydrotalcite. Sensitivity towards potential leachates was quantified through deliberate spiking of our alkali-free Au/HT catalysed oxidations with additional parent Mg–Al HT (pH = 9), or Mg(OH)_2_ (pH = 10) or NaOH (pH 14). Additional HT had negligible impact on FDCA production, while even 3 equiv. of Mg(OH)_2_ only increased the FDCA yield to 38% (Fig. S2†), eliminating the possibility that surface brucite present in high Mg content hydrotalcites could account for the literature value approaching 100% dicarboxylic acid over alkali-precipitated hydrotalcites. In contrast, raising the pH *via* NaOH addition induced a progressive increase in HMF conversion, accompanied by a dramatic switchover in selectivity at pH > 12.5 from HMFCA to FDCA (Fig. 2). *Additional soluble base is thus essential for efficient activation of HMF (and the resultant reactively-formed HMFCA) for this particular HMF* *:* *surface gold ratio*. As we show, later this conclusion cannot be generalised to all substrate : catalyst ratios. A plausible pH dependent reaction mechanism is illustrated in Fig. 2. While not the subject of our present report, we note that the basicity of our alkali-free hydrotalcite is a strong function of thermal processing, with higher temperature calcination or calcination-rehydration treatments^43^ increasing the proportion of strong base sites and corresponding rate of HMF oxidation.

**Fig. 2:**
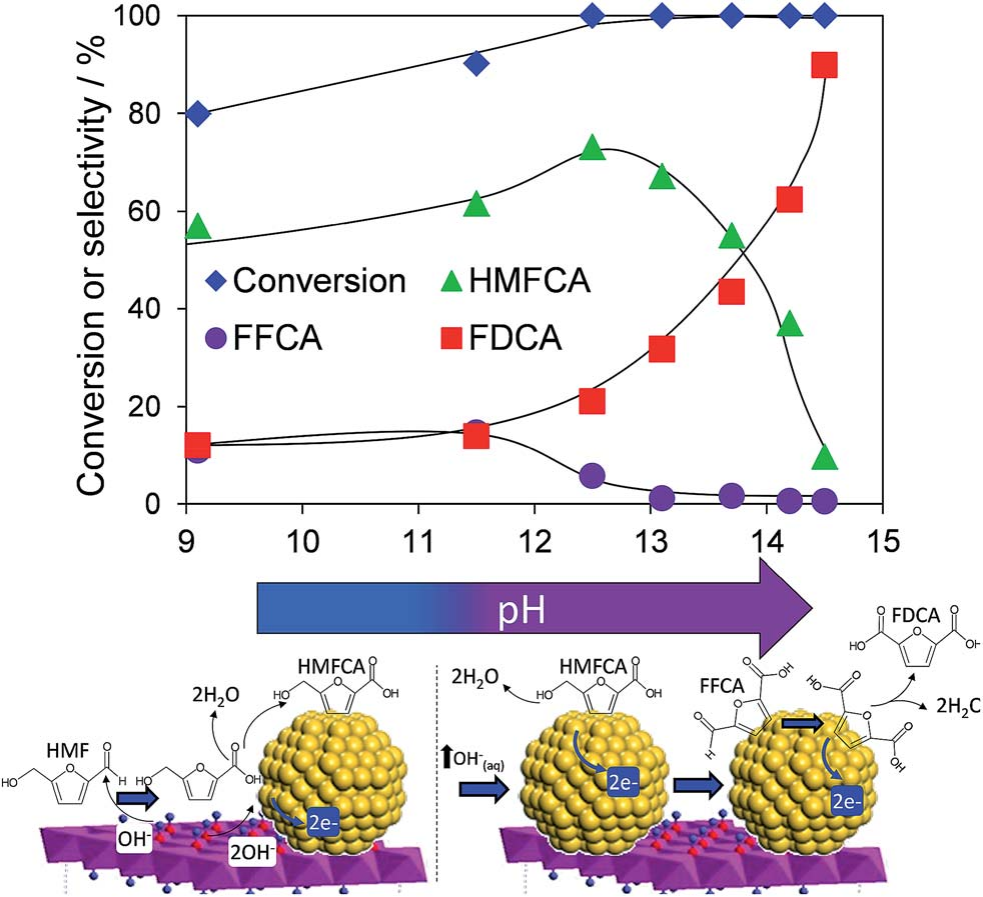
pH dependence of HMF oxidation over a 2 wt% Au/HT catalyst after 7 h reaction, and possible mechanism for surface-initiated HMFCA at pH 9, and solution phase activation at higher pH.

In order to elucidate the origin of this striking pH sensitivity, we have comprehensively mapped the kinetics of individual steps in the reaction pathway with and without NaOH (6 mmol/pH 14). The results are summarised in Scheme 1. In accordance with conventional wisdom, which holds that alcohols are oxidized more slowly than aldehydes over gold,^25^,^48^ HMFCA → FFCA (step 2) exhibited the slowest rate with/without additional base and highest activation energy (40 kJ mol^–1^). However, the aldehyde oxidations in steps 1 and 3 surprisingly exhibited the strongest NaOH dependencies, equating to 100-fold (HMF → HMFCA) and 66-fold (FFCA → FDCA) rate enhancements respectively. These far exceed the comparatively small four-fold enhancement observed for step 2 (HMFCA → FFCA); this appears a general phenomenon for Au catalysed aldehyde *versus* alcohol oxidation (Fig. S3†). We attribute the dramatic impact of soluble base upon HMF oxidation to its consequent displacement of the equilibrium-limited, geminal diol reactive intermediate towards HMFCA;^49^ oxidation of the HMF aldehyde function to the geminal diol is facile, however at low-moderate pH the reverse dehydration is favoured.

**Scheme 1:**
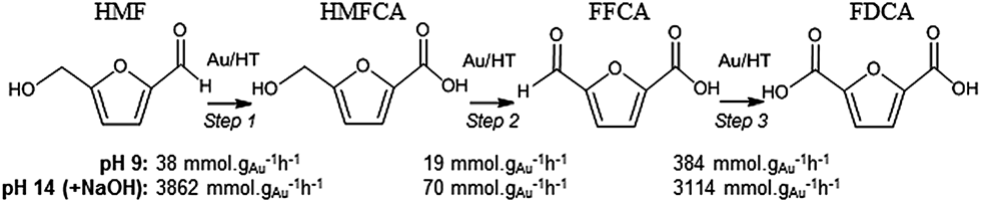
Impact of NaOH on kinetics of HMF oxidation over 2 wt% Au/HT.

The absence of 2,5-dihydroxymethylfurfural (DHMF) indicates that the competing base catalysed Cannizzaro reaction,^50^ wherein the geminal diol disproportionates to HFMCA and DHMF in the solution phase, does not operate in the presence of Au (or that DHMF oxidation is always extremely rapid). HMFCA oxidation is clearly identified as the slowest step in HMF oxidation, but while this step is indeed promoted by soluble base, the greatest impact of NaOH is actually upon HMF oxidation and the attendant increase in HMFCA concentration, a discovery we return to shortly.

The question arises as to nature of the gold active phase under such high pH conditions, and possibility of metal leaching, oxidation or Na–Au intermetallic formation. This was explored through an *operando* XAS study of aqueous phase HMF aerobic oxidation over the preceding 2 wt% Au/HT catalyst at 90 °C (Fig. 3). XANES analysis revealed that gold remained in metallic form before and after NaOH addition, with no evidence for Au(OH)_3_ formation, while EXAFS analysis identified only Au–Au coordination shells with a constant coordination number (Table S1†). These observations confirm that gold nanoparticles do not sinter or leach even after 16 h reaction, and that NaOH directly promotes oxidation without influencing the electronic or structural properties of gold. This is consistent with isotope-labelling and DFT studies,^37^,^49^ which suggest that hydroxyls adsorbed at the edge of Au clusters are the critical surface species participating in the catalytic cycle, lowering the barriers to C–H and O–H dissociation^51^ and removing surface hydride.

**Fig. 3:**
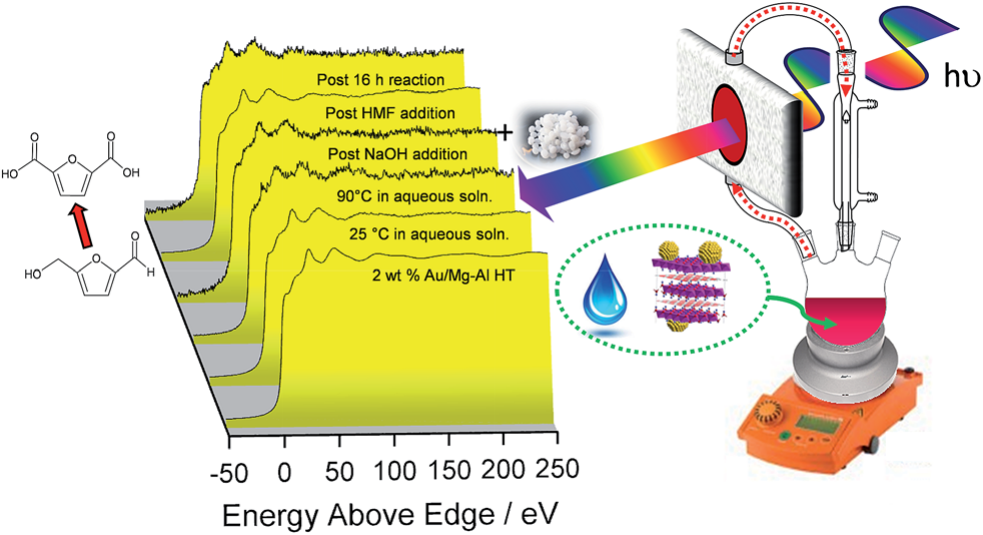
*Operando* Au L_III_-edge XAS of a 2 wt% Au/HT catalyst during aqueous phase selective aerobic oxidation of HMF; catalytically active, metallic gold nanoparticles are unaffected by hot water or NaOH addition.

Fig. 2 demonstrates that alkali-free 2 wt% Au/HT is able to oxidise HMF to FDCA (albeit slowly in the absence of soluble base), while Scheme 1 highlights the principal role of NaOH as to accelerate HMF oxidation to HMFCA, at least partially through suppressing dehydration of the reactively-formed geminal diol intermediate. The latter discovery led us to speculate that the overall oxidation cascade could alternatively be promoted through enhancing the rate of surface catalysed geminal diol dehydrogenation in steps 1 and 3 to corresponding carboxylic acids simply by increasing the Au concentration. A series of Au/HT catalysts were therefore synthesised employing a common, alkali-free Mg–Al HT support with varying gold loadings (Table S2 and Fig. S4–S8†) which exhibited similar solid base strengths and site densities (Fig. S9–S11†). Fig. 4 shows that higher gold loadings indeed promoted FDCA production (at the expense of HMFCA), *with a 78% yield of the desired dicarboxylic acid attainable for 10 wt% Au/HT in the absence of any soluble base*. This largely reflects the ability of gold to ameliorate the strong requirement for additional NaOH to drive the two aldehyde oxidation steps 1 and 3 (HMF → HMFCA and FFCA → FDCA respectively); NaOH rate-enhancements for HMF oxidation fall three-fold as the bulk Au content rises from 0.5–10 wt%, while the sensitivity of the final FFCA oxidation to NaOH falls 40-fold over the same gold range. *High FDCA yields are therefore achievable either by using low concentrations of Au in conjunction with a strong soluble base, or high concentrations of Au on a moderate strength solid base*.

**Fig. 4:**
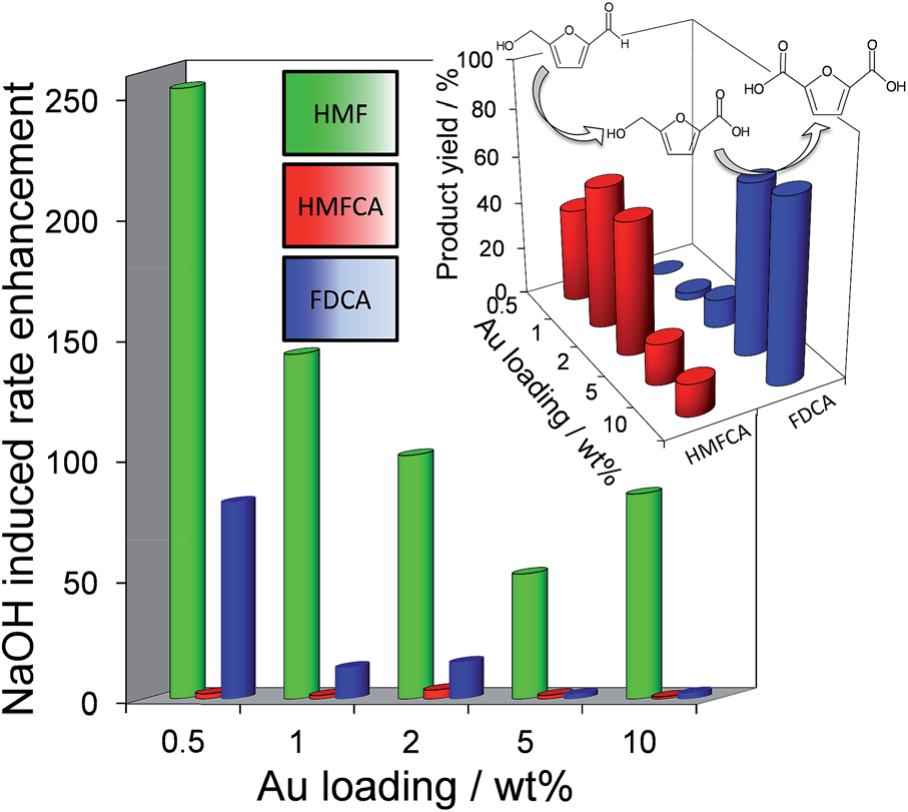
(main) Impact of Au loading on the sensitivity of individual oxidation steps towards soluble base addition over Au/HT catalysts; (inset) Au loading dependent product selectivity in HMF oxidation in the absence of soluble base.

We attribute the loading dependence of these two catalytic regimes (soluble base ≤ 2 wt% Au > solid base) to competitive adsorption between HMF and HMFCA. The HMF : surface Au molar ratio approaches 60 : 1 for the 0.5 wt% Au/HT catalyst, hence it is unlikely that the low concentration of geminal diol formed without NaOH can effectively compete for adsorption sites over gold nanoparticles. NaOH addition accelerates geminal diol formation from HMF in solution, displacing the HMF adsorption equilibrium and liberating reactive gold surface site for both geminal diol dehydrogenation to HMFCA, and subsequent OH^–^ mediated oxidative dehydrogenation of HMFCA → FFCA and FFCA hydration/dehydrogenation to FDCA. In contrast, the HMF : surface Au molar ratio is only 5 : 1 for the 10 wt% Au/HT catalyst, and one may therefore anticipate that the geminal diol faces significantly less competition from HMF for vacant gold sites. These hypotheses are supported by the strong non-linear dependence of FDCA production on HMF conversion (Fig. 5 main). FDCA requires a threshold HMF conversion >80%, indicating that high concentrations of reactively-formed HMFCA from the first oxidation step are necessary to compete effectively with unreacted HMF for subsequent oxidation. In contrast, FDCA production *via* the direct aerobic oxidation of HMFCA is near quantitative and increases linearly with surface Au concentration/conversion, as anticipated for a structure-insensitive reaction in which the reactant coverage is low (weak adsorption or rapid reaction).

**Fig. 5:**
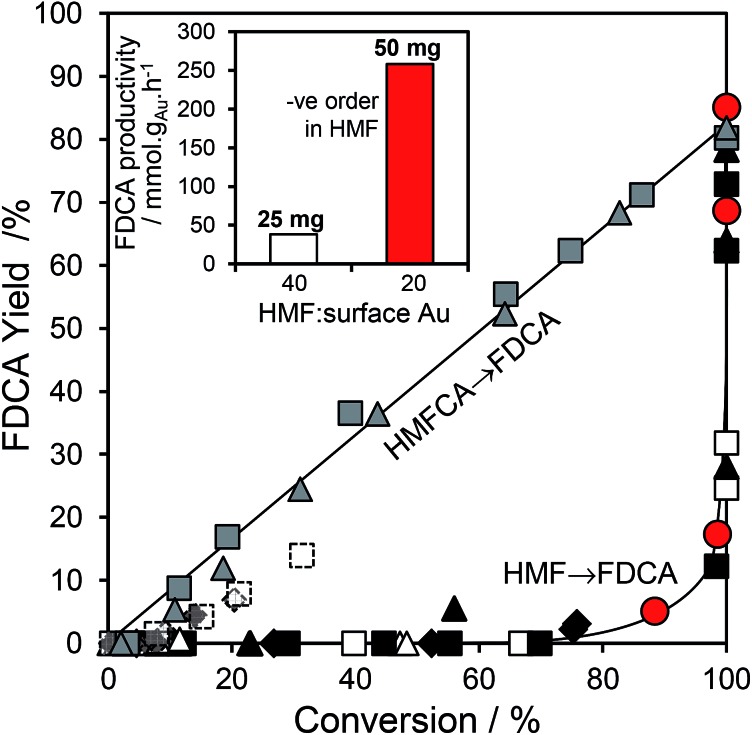
(main) Disparate evolution of FDCA yield as a function of either HMF or HMFCA conversion over 25 mg 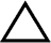
(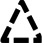) 0.5 wt%, 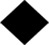
(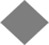) 1 wt%, 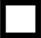
(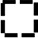) 2 wt%, 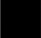
(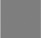) 5 wt%, and 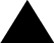
(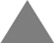) 10 wt% Au/HT catalysts, and 50 mg 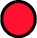 2 wt% Au/HT; (inset) normalised FDCA productivity per Au atom as a function of HMF : surface Au molar ratio for 2 wt% Au/HT highlighting self-poisoning by high HMF concentrations.

Further evidence that strong HMF adsorption site-blocks oxidation of its reactively-formed products is apparent in Fig. 5 inset wherein the HMF : surface Au ratio was varied for the 2 wt% Au/HT catalyst. In the absence of diffusion limitations, and the presence of available reaction sites, the mass normalized FDCA productivity should be independent of substrate : catalyst ratio, whereas Fig. 5 inset reveals that halving the HMF : surface Au ratio imparts a seven-fold increase in FDCA productivity. Reactive gold sites for HMFCA oxidation only become available for HMF : surface Au ratios below a critical threshold wherein it can effectively compete with adsorption of the parent HMF.

In Conclusion, the combination of *operando* XAS and detailed kinetic mapping has elucidated the nature of the active site and mechanism of Au catalysed 5-HMF aerobic selox to 2,5-FDCA. A delicate balance is revealed between the rate of base catalysed 5-HMF activation and the latter's self-poisoning of requisite metallic gold sites for subsequent oxidation of reactively-formed HMFCA/FFCA intermediates. Hydrotalcite solid base can only drive 5-HMF selox in concert with high concentrations of surface gold, a discovery that has important implications for gold catalysis and cascade oxidations.

## Supplementary Material

Supplementary information
