# Supplementary material for: Solid base catalysed 5-HMF oxidation to 2,5-FDCA over Au/hydrotalcites: fact or fiction?
Source: Chem Sci. 2015 Jun 8;6(8):4940–5. doi: 10.1039/c5sc00854a (PMC6088438; doi:10.1039/c5sc00854a)
Supplement: Supplementary file 1 [file SC-006-C5SC00854A-s001.pdf]

Electronic Supplementary Material (ESI) for Chemical Science.
This journal is © The Royal Society of Chemistry 2015

Solid base catalysed 5-HMF oxidation to 2,5-FDCA over Au/hydrotalcites: Fact or fiction?

Leandro Ardemani, Giannantonio Cibin, Andrew J. Dent, Mark A. Isaacs, Adam F. Lee,*
Georgios Kyriakou, Christopher M.A. Parlett, Stephen A. Parry and Karen Wilson*

Structural properties of Au/HT

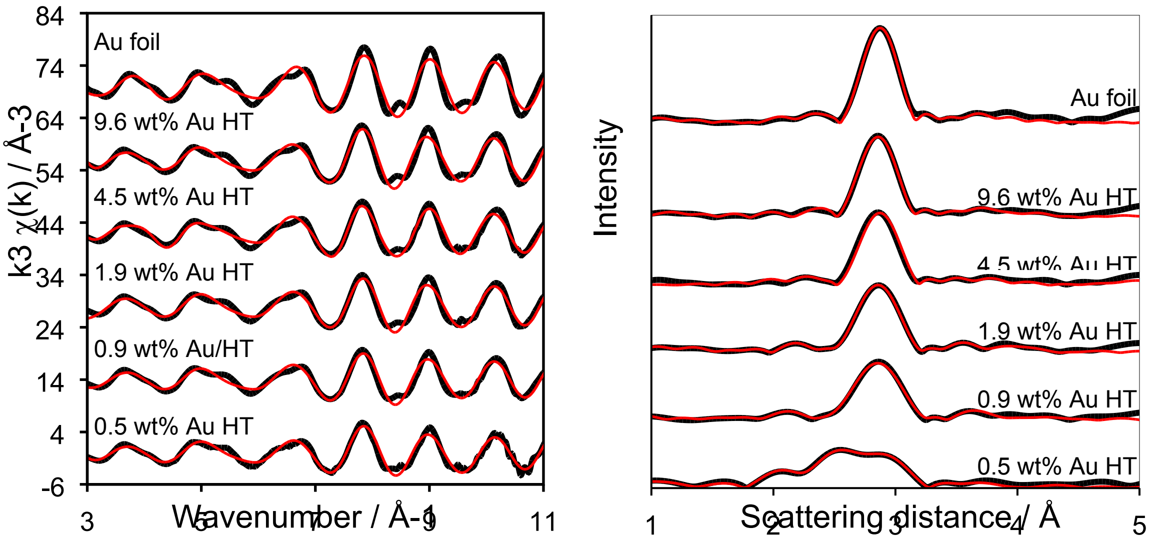

Figure S1. (left) k^3-weighted EXAFS spectra and (right) Fourier transforms of Au/HT catalysts as a function of Au loading.

Table S1. Au L_{III}-edge EXAFS fitting parameters for Au/HT catalysts calcined at 200 °C in air

Au loading / wt%	Scattering pair	Coordination number	Scattering distance / Å	σ / Å²	Fit factor
Au foil	Au-Au	12	2.86 (±0.02)	0.0083 (±0.0005)	2.56
	Au-Au	6	4.04 (±0.03)	0.0159 (±0.0051)	
0.5	Au-Au	10.2 (±0.3)	2.85 (±0.03)	0.0089 (±0.0016)	7.26
0.9	Au-Au	10.5 (±0.2)	2.85 (±0.03)	0.0089 (±0.0010)	6.98
1.9	Au-Au	10.6 (±0.1)	2.85 (±0.03)	0.0084 (±0.0008)	1.94
4.5	Au-Au	11.1 (±0.1)	2.86 (±0.02)	0.0086 (±0.0006)	5.12
9.6	Au-Au	11.4 (±0.1)	2.85 (±0.03)	0.0083 (±0.0006)	7.33

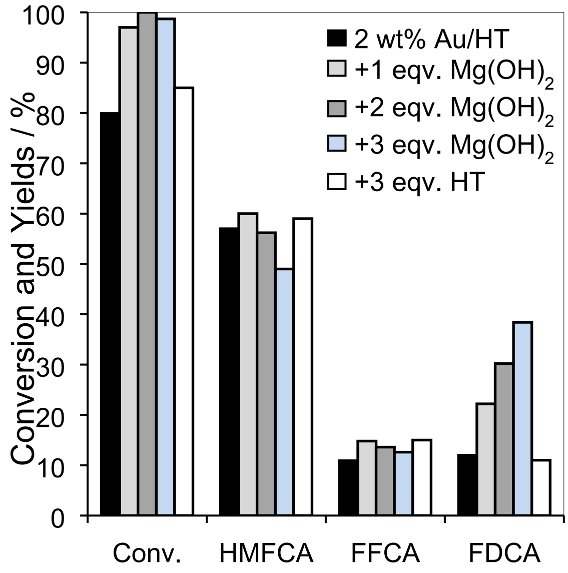

Figure S2. Impact of spiking 2 wt% Au/HT catalysed HMF oxidation reaction with soluble $Mg(OH)_2$ or additional Mg_3Al parent HT.

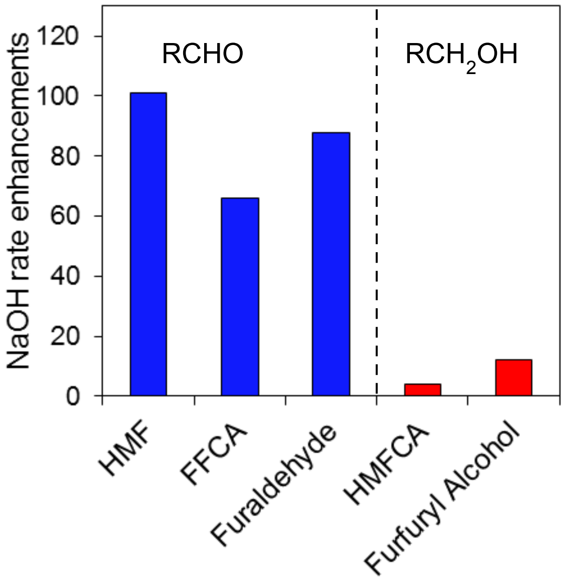

Figure S3. Comparative NaOH rate enhancements for the selective aerobic oxidation of furanic aldehydes versus alcohols over a 2 wt% Au/HT catalyst.

Table S2. Elemental analysis and porosimetry of Au/HT catalysts

Nominal Au loading / wt%	EDX / wt%	ICP / wt%	XPS / wt%	BET surface area / $m^2.g^{-1}$
Parent HT	-	-	-	95
0.5	0.7	0.5	0.3	88
1	1.3	0.9	0.9	88
2	2.6	1.9	1.8	90
5	4.9	4.5	6.9	91
10	9.6	9.6	9.4	75

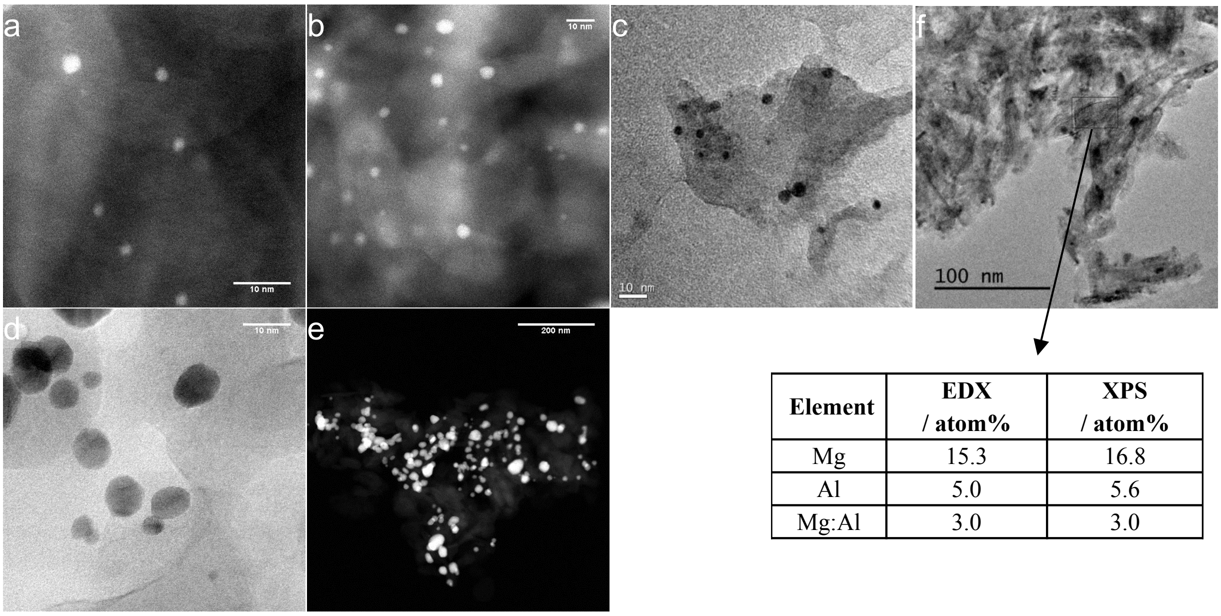

Element	EDX / atom%	XPS / atom%
Mg	15.3	16.8
Al	5.0	5.6
Mg:Al	3.0	3.0

Figure S4. Dark/bright field (S)TEM images of (a) 0.5 wt%, (b) 1 wt%, (c) 2 wt%, (d) 5 wt%, and (e) 10 wt% Au/HT catalysts, and (f) 2 wt% Au/HT showing nanosheets with associated bulk and surface Mg:Al molar composition = 3:1.

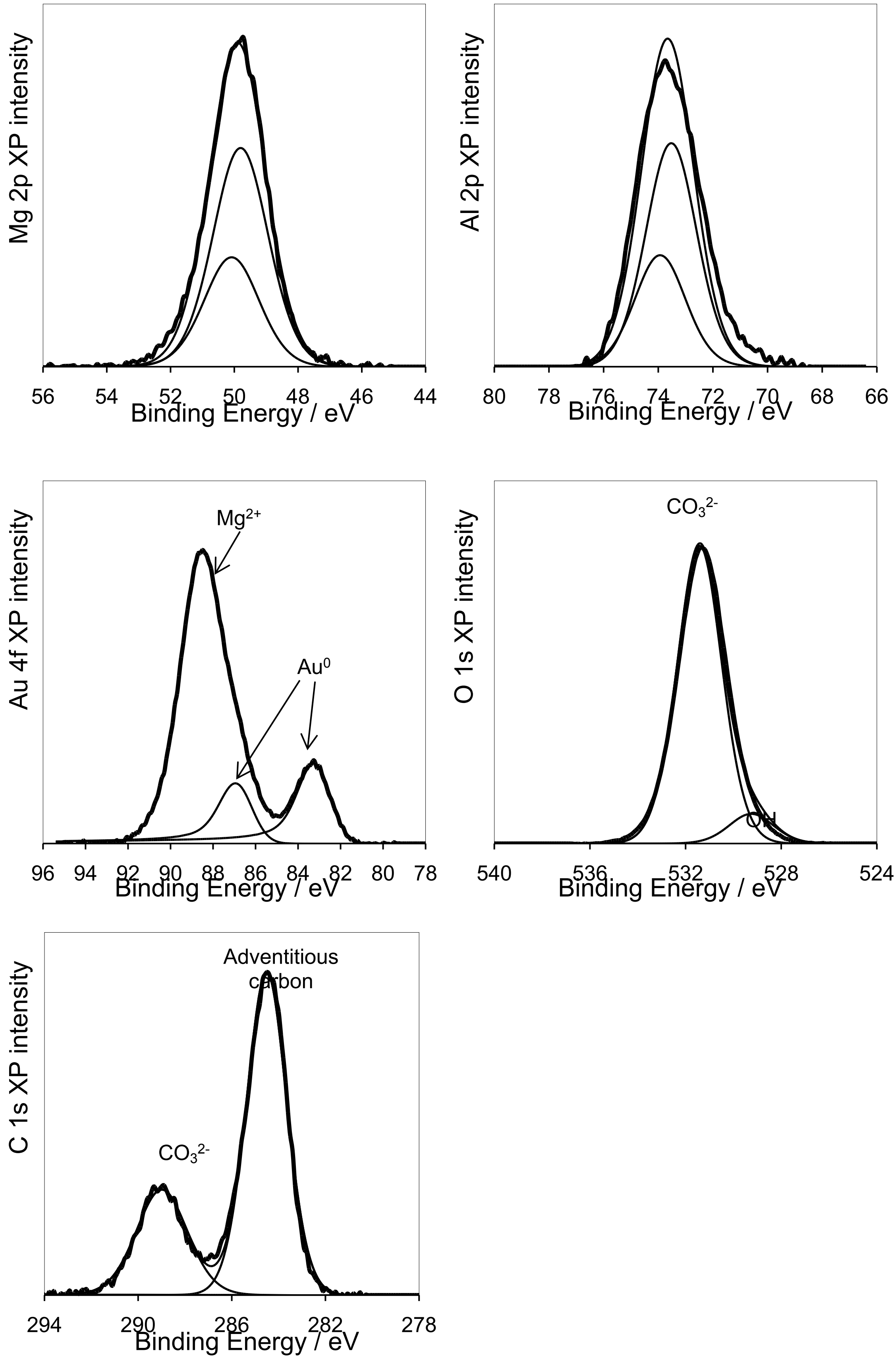

Figure S5. High resolution XP spectra of 2 wt% Au/HT and associated fitted chemical states.

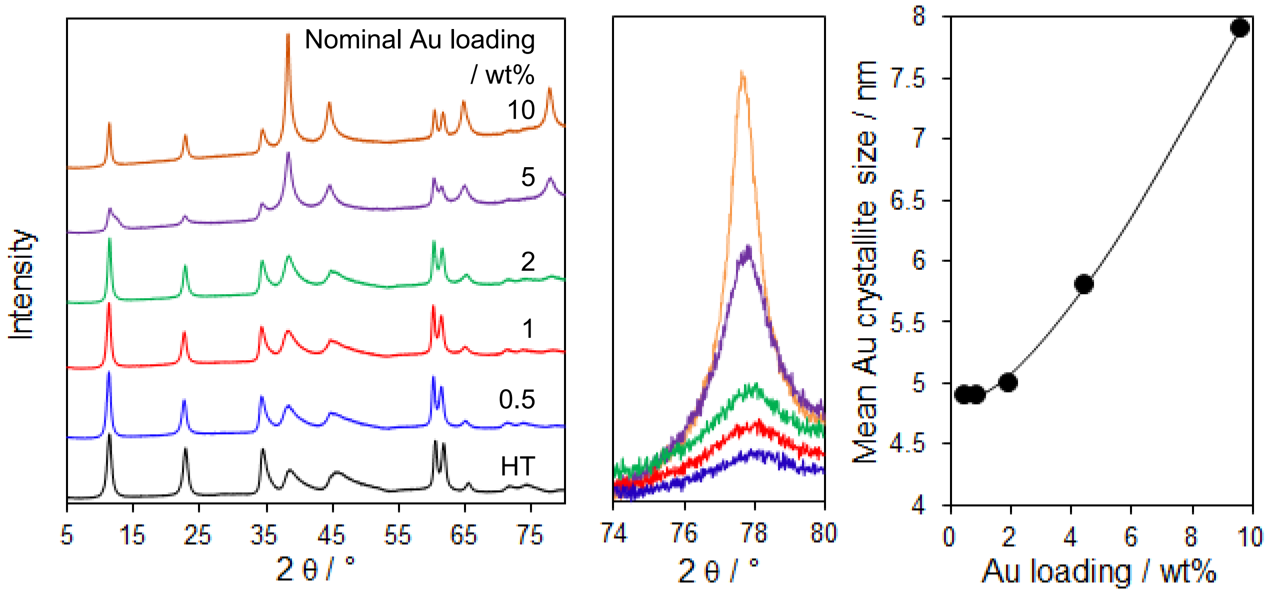

Figure S6. (left) Powder X-ray diffractograms of Au/HT catalysts, and (right) evolution of Au particle size with loading from line broadening of 78 ° reflection by Scherrer analysis.

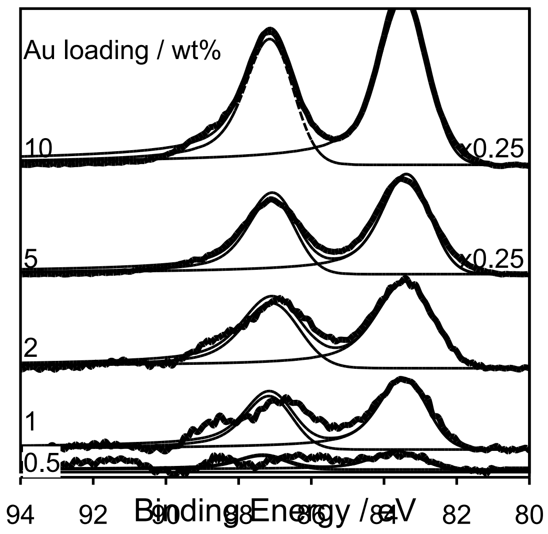

Figure S7. High resolution Au 4f XP spectra of Au/HT catalysts as a function of loading.

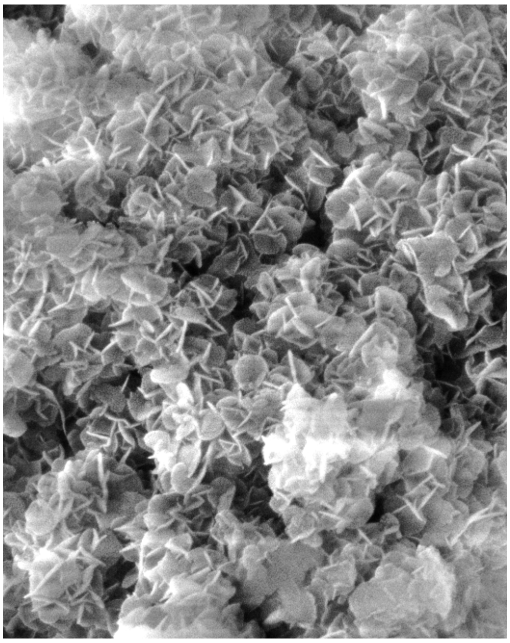

Figure S8. SEM of parent Mg_3Al HT showing characteristic sandrose morphology.

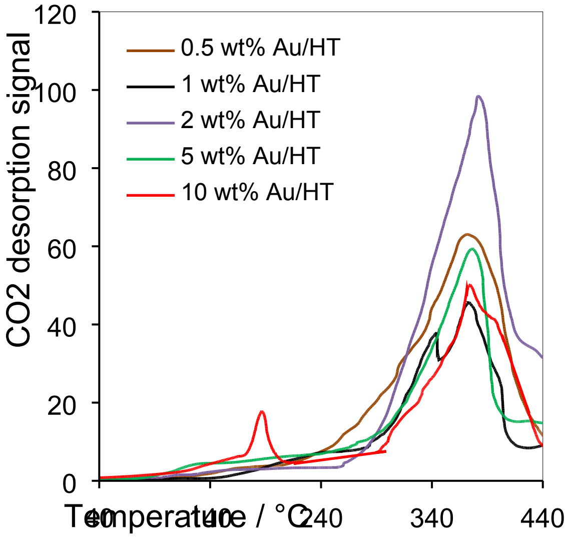

Figure S9. CO_2 temperature programmed desorption profiles SEM of Au/HT catalysts as a function of loading evidencing common medium strength basicity.

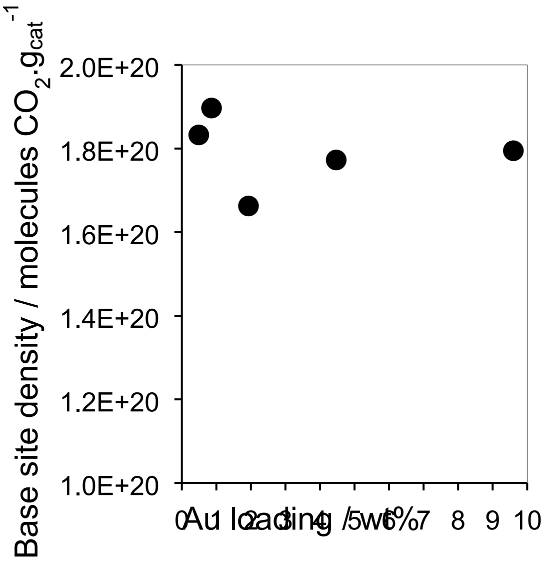

Figure S10. Integrated CO_2 TPD desorption signals of Au/HT catalysts as a function of loading evidencing a common base site density, reflecting their common Mg_3Al HT support.

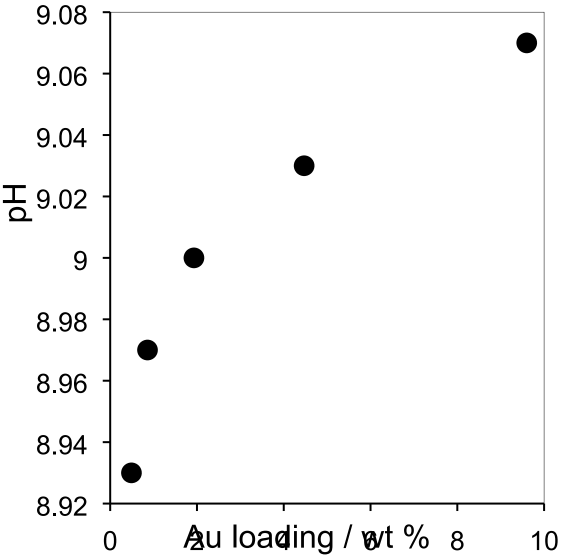

Figure S11. pH dependence in aqueous solution of Au/HT catalysts as a function of loading evidencing a small rise in solution basicity with Au loading

Catalyst synthesis

Alkali-free hydrotalcites were synthesised according to our literature co-precipitation method. A mixture of 1 M $Mg(NO_3)_2.6H_2O$ and $Al(NO_3)_3.9H_2O$ in deionised water was first prepared in a 100 ml volumetric flask at a nominal Mg:Al molar ratio of 3:1. Separately, a 2 M aqueous solution of $(NH_4)_2CO_3$ and NH_4OH of pH 9 was prepared in a second volumetric flasks. The two solutions were simulatneously-fed via an Atlas syringe pump dropwise at room temperature into a 2 L Radleys Ready reactor, filled with 300 ml of de-ionised water, under 300 rpm stirring; the addition rate was set at 1 $ml.min^{-1}$ and the pH maintained between 9.3-9.5 iva addition of concentrated NH_4OH (35 wt.% NH_3 aqueous solution). The resulting white hydrogel was aged overnight at 65 °C, then washed with H_2O until the pH was neutral, and finally dried in vacuo overnight at 100 °C and stored in a dessicator.

Au/HT catalysts were prepared subsequently prepared via deposition-precipitation with nominal gold loadings of 0.5, 1, 2, 5 and 10 wt.%. An appropriate amount of $HAuCl_4$ aqueous solution with [Au] = 10 or 20 $mg.ml^{-1}$, was dissolved in 40 ml H_2O under stirring, prior to the addition of 1 g of dried hydrotalcite. This was followed by the addition of concentrated NH_4OH (35 wt.% NH_3 aqueous solution) until the pH reached 10 (requiring approximately 0.6-0.8 ml of base). The resulting mixture was stirred at room temperature for 6 h, and residual solvent removed under reflux for 30 min, yielding a yellow-orange solid which was filtered and washed with H_2O until neutral. This solid was calcined under flowing O_2 (10 $ml.min^{-1}$) for 4 h at 200 °C, affording a final purple coloured solid, indicative of Au^0 nanoparticles formation.

Catalyst characterisation

Nitrogen porosimetry was undertaken on a Quantachrome Nova 4000e porosimeter; samples were degassed at 120 °C for 2 h prior to analysis, with multi-point BET surface areas calculated over the relative pressure range 0.01-0.3. Powder XRD patterns were recorded on a Bruker D8 ADVANCE diffractometer with a Cu K_α source between 2 θ = 10-80 ° and 0.02 ° step size. XPS was performed on a Kratos Axis HSi X-ray photoelectron spectrometer fitted with a charge neutraliser and magnetic focusing lens employing Al K_α monochromated radiation (1486.7 eV); spectral fitting was performed using CasaXPS version 2.3.15, with spectra enery-corrected to the C 1s peak of adventitious carbon at 284.6 eV. Base site densities were measured via CO_2 pulse chemisorption and subsequent temperature programmed desorption on a Quantachrome ChemBET 3000 system; samples were outgassed at 120 °C under He (120 $ml.min^{-1}$) for 1 h prior to CO_2 titration at 40 °C.

SEM analysis was conducted on an Oxford Instruments EVO SEM; samples were mounted on a carbon disc and coated using 90:10 Au:Pd. EDX analysis was undertaken via Oxford Instruments Inca software on uncoated samples. High-resolution TEM was performed on a Philips EM208 TEM with a tungsten filament at 80 kV. Samples were deposited from ethanolic solution on holey carbon copper grids. Elemental analysis was performed by ICP-MS (MEDAC UK).

Au L_{III} K-edge (11.92 keV) XAS measurements were made on beamline B18 of the Diamond Light Source in fluorescence mode, using a Si(111) double crystal monochromator and 9-element Ge solid state detector. In situ thermal decomposition of the catalyst precursor was followed in a bespoke "Sankar" pellet furnace under flowing air (10 $ml.min^{-1}$) between 25-500 °C. Operando measurements were made in a bespoke PTFE cell with Kapton windows on a catalyst reaction mixture (250 mg of 2 wt% Au/HT, 1 mmol HMF and 60 ml H_2O) recirculated from an external oxygenated round bottom flask (10 $ml.min^{-1}$ flowing O_2 at 1 bar) between room temperature and 90 °C; spectra were processed using Athena and Artemis software within the IFEFFIT software suite.

5-HMF oxidation

Selective oxidation of 5-HMF was conducted in a 3-neck round-bottomed flask using a Radleys Starfish reactor, employing 0.2 mmol HMF in 6 ml of deionised water, and either 25 or 50 mg of Au/HT catalyst under flowing O2 (10 ml.min^{-1}) at 90 °C and 500 rpm stirring (sufficient to eliminate mass-transport effects). Additional 1 M NaOH solution (pH 14) was added to some experiments in order to regulate solution pH as described in the main manuscript.

Aliquots were sampled periodically and analysed on an Agilent Technologies 1200 Infinity HPLC equipped with UV-vis and R.I. detectors. A Zorbax Hilic plus HPLC column (4.6 mm x 100 mm x 3.5µm) was employed, protected by a Rx-SIL guard column (4.6 mm x 12.5 mm), in conunction with a gradient method, developed in co-operation with Agilent Technologies, to achieve resolution of HMF (λ = 282 nm), HMFCA (λ = 260 nm), FFCA (λ = 282 nm) and FDCA (λ = 260 nm). The mobile phase was prepared with acetonitrile as eluent A, and eluent B an HPLC grade aqueous solution buffered by 50 mM CH_3COONH_4; the pH was adjusted to 5.2 by adding the requiste amount of glacial CH_3COOH. The gradient method is defined in Table S3:

Table S3: gradient of eluent for HPLC analysis

Time	% A (CH_3CN)	% B (aqueous buffer, pH 5.2)
0	95	5
5	60	40
6	60	40
6.5	95	5

A typical chromatogram is shown in Fig. S9 highlighting the excellent resolution of all components.

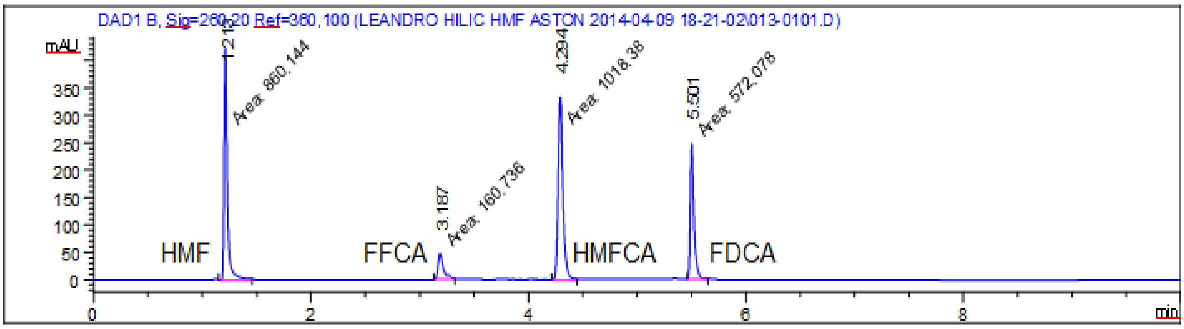

Figure S9. HPLC chromatogram of reaction mixture during 5-HMF oxidation over 2 wt% Au/HT.
